# The highly variable microbiota associated to intestinal mucosa correlates with growth and hypoxia resistance of sea bass, *Dicentrarchus labrax*, submitted to different nutritional histories

**DOI:** 10.1186/s12866-016-0885-2

**Published:** 2016-11-08

**Authors:** François-Joël Gatesoupe, Christine Huelvan, Nicolas Le Bayon, Hervé Le Delliou, Lauriane Madec, Olivier Mouchel, Patrick Quazuguel, David Mazurais, José-Luis Zambonino-Infante

**Affiliations:** 1 NUMEA, INRA, Univ. Pau & Pays Adour, 64310 Saint Pée sur Nivelle, France; 2 Ifremer, UMR 6539 (LEMAR), PFOM/ARN, Centre de Bretagne, CS 10070, 29280 Plouzané, France; 3 PFOM/ARN, Ifremer, Centre de Bretagne, CS 10070, 29280 Plouzané, France

**Keywords:** Host-microbe interaction, 16S rRNA, Pyrosequencing, Autochthonous bacteria, Alternative feed ingredients, Physiological status

## Abstract

**Background:**

The better understanding of how intestinal microbiota interacts with fish health is one of the key to sustainable aquaculture development. The present experiment aimed at correlating active microbiota associated to intestinal mucosa with Specific Growth Rate (SGR) and Hypoxia Resistance Time (HRT) in European sea bass individuals submitted to different nutritional histories: the fish were fed either standard or unbalanced diets at first feeding, and then mixed before repeating the dietary challenge in a common garden approach at the juvenile stage.

**Results:**

A diet deficient in essential fatty acids (LH) lowered both SGR and HRT in sea bass, especially when the deficiency was already applied at first feeding. A protein-deficient diet with high starch supply (HG) reduced SGR to a lesser extent than LH, but it did not affect HRT. In overall average, 94 % of pyrosequencing reads corresponded to Proteobacteria, and the differences in Operational Taxonomy Units (OTUs) composition were mildly significant between experimental groups, mainly due to high individual variability. The highest and the lowest Bray-Curtis indices of intra-group similarity were observed in the two groups fed standard starter diet, and then mixed before the final dietary challenge with fish already exposed to the nutritional deficiency at first feeding (0.60 and 0.42 with diets HG and LH, respectively). Most noticeably, the median percentage of *Escherichia-Shigella* OTU_1 was less in the group LH with standard starter diet. Disregarding the nutritional history of each individual, strong correlation appeared between (1) OTU richness and SGR, and (2) dominance index and HRT. The two physiological traits correlated also with the relative abundance of distinct OTUs (positive correlations: *Pseudomonas* sp. OTU_3 and *Herbaspirillum* sp. OTU_10 with SGR, *Paracoccus* sp. OTU_4 and *Vibrio* sp. OTU_7 with HRT; negative correlation: *Rhizobium* sp. OTU_9 with HRT).

**Conclusions:**

In sea bass, gut microbiota characteristics and physiological traits of individuals are linked together, interfering with nutritional history, and resulting in high variability among individual microbiota. Many samples and tank replicates seem necessary to further investigate the effect of experimental treatments on gut microbiota composition, and to test the hypothesis whether microbiotypes may be delineated in fish.

**Electronic supplementary material:**

The online version of this article (doi:10.1186/s12866-016-0885-2) contains supplementary material, which is available to authorized users.

## Background

There is growing evidence that intestinal microbes play functional roles that are essential to health and nutrition, and a better understanding of the relationship between fish and their gut microbiota is crucial for sustainable aquaculture development [1]. Many factors have been suggested as influencing the origin and composition of gut microbiota of fish, including genetic background [2, 3], diet [4], stress [5], and many environmental factors (e.g., temperature [6]). Regarding European sea bass, *Dicentrarchus labrax*, one of the two main marine fish species produced by aquaculture in southern Europe [7], high similarity was noticed among the faecal microbial communities collected from individuals grouped in the same tank [8]. However, dominant and subordinate individuals of Arctic charr harboured distinct aerobic microbiota associated with intestinal mucosa [9], suggesting that social interaction may affect autochthonous gut microbiota in individuals cohabiting in the same tank. High-throughput sequencing methods have been recently applied to intestinal microbiota in fish. The pyrosequencing of 16S rRNA gene fragments allowed Roeselers et al. [10] to detect bacterial taxa that were shared by the intestinal communities in zebrafish of different origins, including specimens caught in the wild. The authors concluded that fish have a specific core intestinal microbiota, as is the case with higher vertebrates [11]. However, the life history and diet may deeply influence gut microbiome in fish [12].

During the last decade, the composition of fish feeds has considerably changed to find the way to expand aquaculture without depleting natural fish stocks. Fish meal can be almost completely replaced by plant protein sources in the diet of sea bass [13]. The replacement of fish oil by vegetable oils has been also achieved at least partially [14], or in short-term experiments [15], but the high requirement of sea bass for highly unsaturated fatty acids (HUFA) has hindered the simultaneous substitution of fish oil and fish meal by plant ingredients. Scarce information is available about the effect of dietary fatty acids on gut microbiota in fish, and most studies concerned salmonids [4]. Starch and other polysaccharides from vegetal protein sources are known to influence gut microbiota in fish, including sea bass [16].

The composition of starter diets impacted the bacterial community associated with sea bass larvae [17], but the long-term effects of early nutritional treatments on intestinal microbiota have not yet been tested in this species. In rainbow trout fry, a short hyperglucidic hypoproteic dietary stress had short-term and long-term effects on intestinal fungi in juveniles, while intestinal bacteria were not significantly affected [18].

The present experiment was aimed at investigating the short-term and long-lasting effects of dietary stress on gut microbiota in sea bass fed diets either deficient in HUFA, or hypoproteic with starch as substitute for energy supply. To this end, sea bass were challenged with the unbalanced diets, or fed a standard diet, at first feeding (phase 1). After a transition period of 5 months, juvenile fish originating from the different dietary groups were transferred into the challenge tanks for phase 2. This second phase corresponded to a common garden experiment, which mixed in the same tanks animals that were already challenged or not during phase 1. These fish were fed the unbalanced diets for two months, while other individuals fed in standard conditions from start feeding onwards were maintained in a control tank. The physiological status of the individuals was addressed through growth and resistance to hypoxia. These health criteria were compared between dietary groups, and correlated with the composition of the intestinal community of autochthonous bacteria in each individual.

## Results

### Individual variability and dissimilarity of intestinal microbiota

The microbial community was analysed in the intestinal mucosa of 54 fish after two days of fasting at the end of the experiment. The bacterial composition was computed from pyrosequencing data after RNA extraction and reverse transcription, with a view to compare the relative activity of every taxon. The number of valid reads per intestinal sample was highly variable, with significant differences between groups (Table 1). Normalization was thus critical before comparing the rarefaction curves and alpha-diversity. Within each experimental group, a wide variability appeared among samples. The rarefaction curves showed that the number of OTUs was still exponentially increasing even above 11,000 reads in some samples, whereas a rarefaction plateau appeared after much less reads in other ones [see Additional file 1]. Due to individual variability, there was no significant difference between the mean alpha-diversity indices of the experimental groups (Table 1).

**Table 1:** Pyrosequencing yield and mean indices of bacterial diversity in the experimental groups of sea bass

	LH1-LH2	C1-LH2	C1-C2	C1-HG2	HG1-HG2	*p*-value
Valid reads per sample before normalization	17634^ab^ ± 1052	20894^a^ ± 1006	16603^b^ ± 867	18492^ab^ ± 1135	20603^a^ ± 900	0.01
Diversity index after normalization:						
OTU richness	45.6 ± 3.7	50.7 ± 5.1	45.4 ± 3.6	47.3 ± 3.4	49.3 ± 2.9	0.83
Dominance	0.31 ± 0.03	0.38 ± 0.05	0.30 ± 0.03	0.41 ± 0.05	0.33 ± 0.03	0.21
Intra-group similarity (Bray-Curtis)	0.53^ab^ ± 0.02	0.42^c^ ± 0.03	0.52^ab^ ± 0.02	0.60^a^ ± 0.02	0.52^b^ ± 0.02	≤0.001

The means (± SE) were compared by ANOVA; in case of significant difference, superscript letters on the same line indicated the significant differences after post-hoc Tukey’s test. The diversity indices were computed after normalizing the numbers of total reads per sample, based on the minimum observed (11,599 reads). Each group was named after its diets during the two challenge phases: HUFA-deficient diet at both phases (LH1-LH2), or only at phase 2 (C1-LH2); hypoproteic diet with high starch supply at both phases (HG1-HG2), or only at phase 2 (C1-HG2); control with standard diets (C1-C2)

The high variability could be further illustrated by comparing the numbers of OTUs shared among individuals and experimental groups. Over a total of 1111 OTUs, *Escherichia-Shigella* OTU_1 was the only taxon detected in every sample, with a relative abundance varying between 82.5 and 9.6 %. When the experimental groups were compared, 42 OTUs were shared by every group, while 135–159 OTUs were detected only in one group [see Additional file 2].

The intra-group similarity appeared relatively low in the three groups without transfer before the final dietary challenge, with a mean Bray-Curtis index of 0.52–0.53 (Table 1). Interestingly, the same index was differentially affected when the fish were transferred to the challenge tanks, depending on the diet. The individuals submitted to HUFA restriction only during phase 2 presented the lowest intra-group similarity in gut microbiota composition (group C1-LH2; mean Bray-Curtis index: 0.42; difference significant with every other group), while the highest similarity was observed among the fish transferred to the tank challenged with the starchy hypoproteic diet (group C1-HG2; mean Bray-Curtis: 0.60; difference significant only with groups HG1-HG2 and C1-LH2).

Despite individual variability, the non-parametric multivariate analysis of variance (PERMANOVA) revealed an overall significant difference between the mean bacterial profiles of the experimental groups, but none of the Bonferroni-corrected *p* values was less than 0.05 in the post-hoc pairwise comparison (Table 2). The overall significant difference was mainly due to the dissimilarity between the two groups submitted to HUFA deficiency, and between the control group reared in standard conditions (C1-C2) compared to each of the two groups transferred for phase 2.

**Table 2:** Comparison of inter-groups similarities in gut microbiota composition

PERMANOVA between all groups (*p*-value = 0.02)					
Group	LH1-LH2	C1-LH2	C1-C2	C1-HG2	HG1-HG2
LH1-LH2		*0.23*	*1*	*1*	*1*
C1-LH2	0.02		*0.07*	*1*	*1*
C1-C2	0.30	0.007		*0.20*	*0.64*
C1-HG2	0.21	0.15	0.02		*1*
HG1-HG2	0.47	0.18	0.06	0.65	

PERMANOVA analysis on Bray-Curtis distance (9999 permutations); post-hoc pairwise comparison: uncorrected (left) and Bonferroni-corrected *p* values (right, in italics); each group was named after its diets during the two challenge phases: HUFA-deficient diet at both phases (LH1-LH2), or only at phase 2 (C1-LH2); hypoproteic diet with high starch supply at both phases (HG1-HG2), or only at phase 2 (C1-HG2); control with standard diets (C1-C2)

### Phylogenetic analysis of intestinal microbiota

The dominant phylum was Proteobacteria in every sample (94.4 ± 1.0 %), with mainly Gammaproteobacteria (56.5 ± 2.6 %) and Alphaproteobacteria (35.0 ± 2.9 %). Three other phyla were significantly represented: Bacteroidetes (2.3 ± 0.5 %), Actinobacteria (1.4 ± 0.3 %), and Firmicutes (1.1 ± 0.3 %). At the phylum level, the only significant difference after ANOVA corresponded to Actinobacteria, which were more abundant in group C1-C2, compared to C1-HG2, but some other differences were observed between groups after Linear discriminant Effective Size (LEfSe) pairwise analysis [see Additional file 3].

**Fig. 1:**
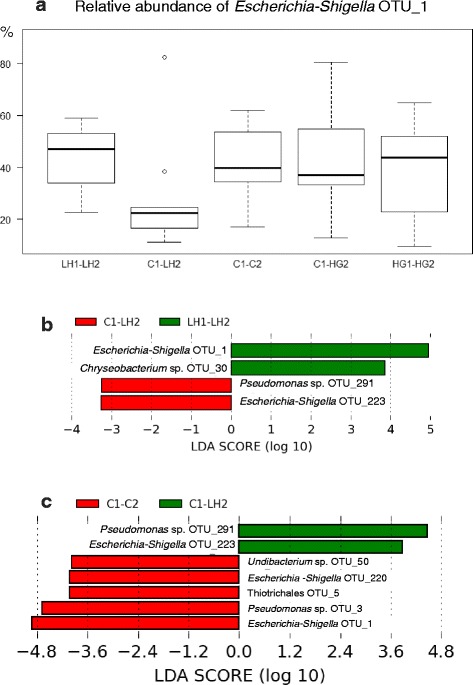
OTU dissimilarity between the two groups submitted to HUFA deficiency, compared to the control group. *Legend:* When all the experimental groups were compared by ANOVA, the high individual variability masked the significance of the relatively low proportion of *Escherichia-Shigella* OTU_1 in group C1-LH2, fed the HUFA-deficient diet only during the final challenge (**a**). However, the LEfSe pairwise comparisons showed significant Linear Discriminant Analysis (LDA) scores when C1-LH2 was compared to the other HUFA-deficient group LH1-LH2 (**b**), and to the control group C1-C2 (**c**). The few other significant LEfSe differences between OTU abundances were also shown in diagrams **b** and **c**

The proportion of 41 OTUs exceeded 5 % in at least one sample, with great variability among individuals [see Additional file 4]. At the OTU level, the most noticeable difference between groups concerned *Escherichia-Shigella* OTU_1, which was generally dominant, but significantly less prevalent in group C1-LH2 compared to group LH1-LH2 or C1-C2 by LEfSe, though there was no significant difference after ANOVA on every group, due to high individual variability (Fig. 1).

*Pseudomonas* OTU_3 was the only major OTU with a significant difference detected by ANOVA, with a mean relative prevalence of 14.7 % in the control group C1-C2, which represented more than the double of the mean proportions in the other groups. The hypoproteic diet with high starch supply had little effect on gut microbiota composition, which appeared rather similar between the two groups, with or without dietary challenge at first feeding. The most noticeable difference concerned *Alkanindiges* sp. OTU_14, which was not detected in group C1-HG2 [see Additional file 5].

There were other significant differences between groups at every phylogenetic level, as revealed by ANOVA, Kruskal-Wallis or LEfSe. It concerned some Gammaproteobacteria [see Additional file 5], Alpha- and Beta-Proteobacteria [see Additional file 6], Actinobacteria and Firmicutes [see Additional file 7], Bacteroidetes and Spirochaetae [see Additional file 8].

However, some of these differences appeared more visible between individuals than between groups. For example, three distinct OTUs of *Bacillus* sp. were relatively active, each one in three different samples from group C1-LH2 (OTU_178, OTU_196, and OTU_202, accounting for 1.2, 0.8, and 0.6 % of total reads in each of the three samples, respectively), while this genus was not detected elsewhere, except at a low level in one sample from group C1-HG2 (OTU_202, 0.05 % total reads).

### Growth and resistance to hypoxia of sea bass

The highest mean weight corresponded to group HG1-HG2 at the three sampling dates, even at the beginning of phase 2 at 225 dph [see Additional file 9]. At this date, the mean weights of groups C1-C2, C1-LH2 and C1-HG2 were significantly lower than that of HG1-HG2. At 266 and 287 dph the mean weights of LH1-LH2 and C1-LH2 were significantly lower than that of HG1-HG2. The Specific Growth Rate (SGR) during this last three weeks was chosen as the best growth indicator, with a view to reduce the incidence of the initial differences in mean weights at the beginning of phase 2. During the final three weeks, the highest mean SGR was observed in the control group C1-C2 (Table 3). The lowest SGR were observed in the groups fed the HUFA-deficient diet, especially when the dietary challenge was applied at both phases (group LH1-LH2). The hypoproteic diet with high starch supply resulted in mild SGR.

**Table 3:** Specific Growth Rate (SGR) and final Hypoxia Resistance Time (HRT) of sea bass

Experimental group	SGR	HRT (h)
LH1-LH2	0.94^d^ ± 0.03	6.63^c^ ± 0.08
C1-LH2	1.05^c^ ± 0.03	6.78^bc^ ± 0.07
C1-C2	1.29^a^ ± 0.02	6.96^ab^ ± 0.07
C1-HG2	1.16^b^ ± 0.03	7.20^a^ ± 0.07
HG1-HG2	1.14^b^ ± 0.02	7.03^ab^ ± 0.07

The individual specific growth rate (SGR) was computed during the last 3 weeks of the dietary challenge. The means (± SE) without common superscript letter on the same column corresponded to significant differences according to the post-hoc pairwise comparisons after ANOVA (*p* ≤0.001, Tukey’s test) and Kruskal-Wallis test (*p* ≤0.001, Dunn’s test) for SGR and HRT, respectively. Each group was named after its diets during the two challenge phases: HUFA-deficient diet at both phases (LH1-LH2), or only at phase 2 (C1-LH2); hypoproteic diet with high starch supply at both phases (HG1-HG2), or only at phase 2 (C1-HG2); control with standard diets (C1-C2)

At the end of the experiment, the group fed HUFA-deficient diet at both periods was significantly less resistant to hypoxia than those with normal HUFA dietary supply. The two groups that were fed unbalanced diets only during the last two months presented also a significant difference in mean Hypoxia Resistance Time (HRT), while the other pairwise comparisons did not indicate further significant differences (Table 3).

### Correlation between gut microbiota composition and host’s growth or resistance to hypoxia

**Fig. 2:**
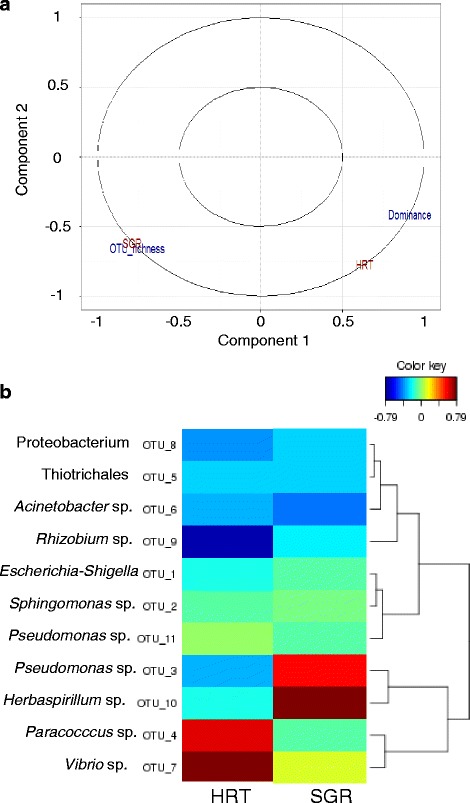
Correlations between physiological traits and characteristics of gut microbiota, whatever the rearing history. *Legend:* **a** Partial Least Square (PLS) canonical correlation between SGR and HRT with alpha-diversity indices in gut microbiota (OTU richness and dominance). The projection vectors on the circle plot reached the maximum score for the four variables, which strongly correlated by pairs. Both pairs of vectors were almost perpendicular, showing the independence of the two variables in each matrix. **b** Heatmap of the sparse PLS canonical correlation between SGR and HRT with the 11 most abundant OTUs in gut microbiota, each one representing more than 0.5 % of total reads. The analysis was based on three components, taking into account all of the 11 abundance scores and the two physiological traits. The *red-brown* and *deep blue* colours indicate strong positive and negative correlations, respectively (correlation coefficient between the two variables ranging from 0.6 to 0.79 in absolute value, see the colour key scale)

Due to some missing data, the complete correlation analysis could be performed only with 51 fish [see Additional file 10]. The individual data of SGR and HRT were put in front of the two indices used to describe alpha-diversity in gut microbiota. The means of OTU richness and dominance were not significantly different between experimental groups (Table 1), but disregarding the groups, the individual scores appeared dependent on the physiological traits. The Partial Least Squares (PLS) analysis showed that SGR correlated strongly with OTU richness, whereas HRT correlated with dominance (Fig. 2a). The two physiological traits correlated also with the relative prevalence of two distinct sets of OTUs (Fig. 2b). SGR correlated with the prevalence of *Herbaspirillum* sp. OTU_10 and *Pseudomonas* sp. OTU_3 (correlation scores around 0.8 and 0.6, respectively), while HRT correlated positively with *Vibrio* sp. OTU_7 and *Paracoccus* sp. OTU_4, but negatively with *Rhizobium* sp. OTU_9 (correlation scores around 0.8 and 0.6, and −0.8, respectively). The correlation between SGR and *Pseudomonas* sp. OTU_3 could be partly explained by the higher prevalence of the bacterium in the fast-growing control group, but the four other correlations seemed independent from the experimental conditions.

## Discussion

There is growing evidence indicating the impact of dietary components on fish gut microbiota, which seems essential for host health and well-being [4]. However, apparent contradiction may arise between some observations obtained under different conditions. The present experiment attempted to evaluate the short and long-term influence of two kinds of nutritional deficiencies on the bacterial community associated with intestinal mucosa in sea bass individuals. When animals are subjected to nutritional stress, not all individuals may react in a similar way, partly depending on intestinal microbiota [19]. A common garden approach was used to test the effects of possible social interaction and inter-individual contamination.

High-throughput sequencing has notably changed the insight on intestinal microbiota in fish, including European sea bass, which has been recently studied with such methods. Carda-Diéguez et al. [20] identified around 78 bacterial families in autochthonous intestinal microbiota of sea bass from genomic DNA, which was in the same range as the 90 families detected in the present dataset obtained from cDNA. However, the phylogenetic profiles were quite different with large proportions of Bacteroidetes, Tenericutes, and Firmicutes in the fresh samples analysed in the previous study. Surprisingly, after storage at −80 °C, Carda-Diéguez et al. [20] reported also a shift of the bacterial profile towards an overwhelming dominance of Proteobacteria. The main genera were not the same as in the present dataset, also dominated by Proteobacteria, but where *Ralstonia* sp. and *Methylobacterium* sp. accounted only for 0.05 and 0.02 % of total reads, respectively, and where *Bradyrhizobium* sp. was not even detected. A large drift of bacterial profiles during cold storage was unlikely in the present study, as the samples were immediately soaked in RNAlater before deep-freezing. Another potential source of bias may be due to resorting to nested PCR for mucosal samples with low concentration of bacterial 16S rRNA. Yu et al. [21] expressed reservation about the detection of rare OTUs after using nested PCR. The present analysis focused on the most active OTUs, which were likely the most susceptible to dietary influence. For this reason, the dataset was limited to the 1111 OTUs that represented at least 0.0002 % of total reads. The most prevalent OTU was the only taxon shared by every individual, but this did not necessarily contradict the core theory applied to fish intestinal microbiota [10], as the rare or weakly active bacteria could not be detected with the present method.

A striking feature of the present data was the high variability of the bacterial profiles among individuals reared in the same conditions. This may also happen in wild fish living in the same environment. Star et al. [22] displayed heterogeneous pyrosequencing profiles among the intestinal contents of 11 specimens of Atlantic cod, which were caught in one location, and then kept in a common tank for seven to twelve days of fasting. These results confirmed that gut microbiota composition may keep original features in fish individuals, even after mix in the same environment, in the absence of feed supply, which could blur the analysis done from intestinal contents. Some individual variation was already observed in the faecal microbiota from European sea bass, but the intestinal bacterial profiles of fish confined in the same tank were much more similar than those reared in replicate tanks [8]. The study was based on the analysis of faeces that were emitted during the overnight isolation of individuals in separate aquaria, which led to conclude to a close intra-tank similarity between individual samples. After release, faecal microbiota evolved under the influence of nutrients and aquatic environment. Faeces collected after some delay cannot accurately reflect the intimate interaction between host intestinal mucosa and associated microbiota. Before comparing different microbial datasets, it is essential to distinguish the nature of the samples, which may come from faeces, digestive tract contents, or intestinal mucosa. The transient bacterial populations in the intestinal content (“allochthonous”) are known to differ from the community adhering to mucus in fish intestine (“autochthonous”) [23–25]. In the autochthonous community of sea bass intestine, Carda-Diéguez et al. [20] noted a higher diversity than in the samples of intestinal content, which appeared less reliable and less representative of the complexity of the bacterial consortium living in fish intestine. This later result confirmed the interest to focus on mucosal samples when studying the interaction between gut microbiota and the host, whereas the analysis of intestinal contents would be more dependent on the interaction with the diet.

Some relationship between individual characteristics and intestinal microbiota were reported in the literature. For example, the distribution of cultivable bacteria differed between the intestinal samples of dominant and subordinate Arctic charr [9]. Two classes of orange-spotted with slow and fast growth rates presented two distinct bacterial profiles [26]. Similar observations were done on Atlantic cod larvae, and less clearly on mangrove killifish larvae [3]. Sea bass can also exhibit dominant behaviour, leading to different individual growth rates, which may account partly for the variability in intestinal microbiota, besides genetic factors. In flow-through water systems, there may be cross contamination between the bacterial communities in fish intestine, fish skin, and those adhering to tank wall. Microbiota associated with cutaneous mucus was influenced by the diet in Atlantic salmon [27], and by genetic background in brook charr [28]. The interactions between fish individuals and bacterial communities within each tank may therefore cause partly the dissimilarity that was observed between intestinal microbiota collected from different tanks. The lack of replication of the experimental treatments in several tanks limited thus the conclusiveness about the effects of the diets in the present study. Some mean proportions of OTUs in the control group were significantly different from others in groups treated with a deficient diet, but it cannot be excluded that these differences might be due to tank cohabitation, rather than actually due to the diet (e.g., *Pseudomonas* sp. OTU_3).

The maintenance of discernible characteristics in gut microbiota composition after three months of common garden in fish individuals with two different nutritional histories was an innovative finding. The evidence came from the tank subjected to HUFA restriction during the final two months of dietary challenge, which was applied to fish either reared previously in standard conditions, or already deprived from normal HUFA supply at first feeding. Ringø et al. [29] “suggested that dietary fatty acids affect the attachment sites for the gastrointestinal microbiota, possibly by modifying the fatty acid composition of the intestine wall”. Dietary HUFA are known to influence the immune status of sea bass [30], and that might also impact the association of bacteria to intestinal mucosa. Bacterial colonization of marine fish larvae may be affected by dietary fatty acids [31], but the hypothetical long-term effect of such initial colonization remains to be investigated in fish. However, the restriction of dietary HUFA at start feeding compromised durably the growth potential of sea bass, as indicated by the lower SGR observed in the individuals already challenged during the larval stages, compared to those submitted to similar deficiency only during the final period. The long-term effect of the initial deficiency might also undermine the development of the immune system and the cell-wall defences in the intestine, which could explain why gut microbiota remained dissimilar between the two groups in the same tank. The most visible differences between these two groups lied in the highest individual variability and in the lowest average proportion of *Escherichia-Shigella* OTU_1 that were observed in the group transferred from the control tank for feeding the HUFA-deficient diet in phase 2. The high individual variability can be perceived through the diversity of the prevalent OTUs that competed with OTU_1 in the latter group, though their average proportions did not result in significant differences between groups. In the other groups, the apparent similarity of the relative proportion of OTU_1 might mask possible differences in bacterial load, especially when comparing the control group to those submitted to HUFA deficiency.

Besides HUFA, other feed components could affect the host-microbe interaction. In particular, the proportion of lupin meal was much higher in the HUFA-deficient diet than in the two others diets of the final challenge (Table 4). This alternative protein source is rich in non-starch polysaccharides, and it influenced gut microbiota composition in sea bass [16]. It might induce the relatively high activity of *Bacillus* sp. in some samples, as these OTUs were close to strain DFEL3.4, previously isolated from the stomach of gilthead sea bream fed lupin meal in the same laboratory [32] [see Additional file 11].

**Table 4:** Composition of the experimental diets

Ingredients (g kg^−1^)	Larval conditioning diets (phase 1)			Nutritional challenging diets (phase 2)		
(Dry matter basis)	LH1	C1	HG1	LH2	C2	HG2
Fish meal	70	520	300	200	350	290
Defatted fish meal	400	–	–	–	–	–
Fish soluble	150	150	150	80	150	90
Soft white lupin	–	–	–	520	300	240
Marine phospholipids	–	–	20	–	–	–
Fish oil	–	–	–	–	–	10
Rapeseed oil	–	–	–	80	80	80
Soybean oil	40	–	–	–	–	–
Rapeseed lecithin				30	30	30
Soy lecithin	200	200	200			
Starch	–	–	210	70	70	240
Vitamin mix	80	80	80	10	10	10
Mineral mix	40	40	40	10	10	10
Cellulose	20	10	–	–	–	–
Proximate composition (%, dry matter basis)						
Crude protein (N × 6.25)	43.5	46.7	32.0	44.8	53.0	40.3
Crude lipids	28.4	23.1	22.1	15.0	13.7	13.2

The hypoproteic diet with high starch supply had little effect on gut microbiota composition. This was different from the results obtained previously in sea bass fed isoproteic diets with either lupin meal, or starch, or cellulose as carbohydrate source [16]. The impact of the diet on gut microbiota was likely moderated in the present experiment by introducing lupin meal in every diet during the final challenge.

Beyond the experimental treatments, the correlative study allowed to highlight the interaction between gut microbiota composition and individual characteristics of the host, probably depending on genetic background and/or traits of behaviour. Such relationship should be carefully interpreted, as the correlation network is likely intricate. However it seems possible to associate physiological traits such as growth potential and hypoxia resistance to some characteristics of gut microbiota in fish, as already attempted between quantitative trait loci and specific bacterial strains associated with the skin of brook charr [28]. Using DGGE, Forberg et al. [3] noted higher band richness in large killifish larvae compared to small individuals, but Shannon index and evenness were not significantly different, whereas the reverse observations were done with Atlantic cod larvae. Pyrosequencing allowed much deeper insight into bacterial diversity than DGGE, and the dominance index appeared clearly independent from OTU richness, which strongly correlated with SGR in sea bass. That suggested that the gut microbiota of fast-growing individuals might be more flexible, due to the presence of numerous taxa, which were not necessarily prevalent, but which could be activated in response to environmental changes, possibly benefiting to the host. The relationship between fish growth and gut microbiota may however depend on rearing conditions, especially on those aimed at managing the intestinal community. A synbiotic treatment with a probiotic strain of *Lactococcus lactis* and oligosaccharides increased growth in Siberian sturgeon, while decreasing gut microbial richness and Shannon index [33]. More surprising was the strong correlation between the dominance index in gut microbiota and the host’s capacity to resist hypoxia. It is admitted in fish as in other vertebrates that the resistance to hypoxia depends on several physiological traits including strong capacity for metabolic depression and high energy reserves to fuel anaerobic metabolism [34, 35]. A longer exposure to hypoxia might favour the relative prevalence of *Vibrio* sp. OTU_7 in some resistant individuals, possibly by stimulating specific metabolic reactions as in *Vibrio cholerae* [36–38]. However, the fish were exposed to hypoxia for 6–8 h, and this range of variation seemed rather short to affect differentially intestinal microbiota. The diet might interfere and, as contrary to lupin meal, dietary starch stimulated *Vibrio* spp. in autochthonous gut microbiota of sea bass [16]. In the present experiment, the HUFA-deficient diet, which depressed HRT, was rich in lupin meal with limited starch supply. Circumspection was nevertheless required to interpret partial least square correlation between HRT or SGR and the most prevalent OTUs, which were highly variable among individuals. For example, the negative correlation between HRT and *Rhizobium* sp. OTU_9 seemed mainly due to one individual, which showed the lowest HRT (6.04 h) and the highest percentage of OTU_9 (25.4 % total reads), whereas this OTU was detected only in seven individuals.

## Conclusion

The main lesson is that active microbiota associated with intestinal mucosa may considerably vary among sea bass individuals, and large samples collected in several replicate tanks will be necessary to attempt at understanding the possible roles of gut microbiome. The severe HUFA restriction highlighted the interference between fish phenotype and gut microbiota, showing that its variability is not merely stochastic, but linked to life history or genetic background. This may suggest to investigate whether microbiotypes could be delineated in fish as in mammals [39], likely in terms of bacterial functions, rather than phylotypes.

## Methods

### Fish and rearing protocols

**Fig. 3:**
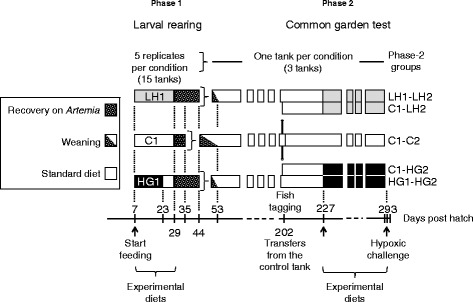
Rearing history, distribution scheme, and experimental schedule of sea bass. *Legend:* In phase 1, the larvae were challenged at first feeding with deficient diets LH1 or HG1, compared to standard diet C1 (Table 4), and then reared in standard conditions. After tagging, some juveniles were transferred from the control group to two experimental tanks for a common garden test in cohabitation with other individuals submitted to nutritional deficiency in phase 1. In phase 2, the juveniles were challenged again for two months with diets LH2 or HG2, compared to C2 (Table 4), and then exposed to a final standardized hypoxic test, 24 h before sampling

Newly-hatched sea bass larvae were provided by Aquastream (Ploemeur, France), allotted at 2 dph (day post hatch) in 15 tanks of 35 L, and then reared in the general conditions described elsewhere [40]. The 15 tanks were divided in three groups of five tanks, which were fed the compound diets from 7 dph onwards (Fig. 3). Two unconventional diets, LH1 and HG1, were tested in comparison with a control diet C1 (Table 4). Diet LH1 consisted in low HUFA content by using defatted fish meal and soybean oil (less than 0.3 % EPA + DHA, dry matter basis). This diet was administered for the 22 first days of feeding, till 28 dph. Starch replaced 40 % of fish meal in the hypoproteic and hyperglucidic diet HG1, which was used from 7 to 22 dph, and then the larvae of this group were fed diet C1 till 28 dph. At 29dph, all the larvae were fed *Artemia* nauplii, with a view to compensate for the growth deficit caused by the deficient diets. At 35 dph, the larvae of the control group, fed diet C1 from 7 to 28 dph, were big enough to be grouped together in one 450-L tank (10,014 fish in total from the five replicated tanks), and then progressively weaned onto standard diet from 35 to 52 dph. Totals of 3662 and 6269 larvae previously fed LH1 and HG1, respectively, were transferred to two other 450-L tanks at 44 dph, and then progressively weaned onto standard diet till 52 dph. The water temperature was maintained at 20 °C during all the rearing period, in an open system without recirculation. At 191 dph, as the juveniles grew up, 500 individuals were randomly selected from each of the three dietary groups C1, LH1 and HG1 (individual mean weight of 16.6, 16.7, and 15.4 g, respectively). At 202 dph, a PIT-tag (PIT: passive integrated transponder) was subcutaneously implanted in every fish that was selected for the second phase of the experiment. Five groups of 100 tagged individuals were named after the diets fed during the two experimental phases (Table 4). The groups were formed as follows: three lots were randomly selected from the initial group fed diet C1, one still fed control diet (final group C1-C2), while the two other groups were mixed either with fish from group LH1 or HG1 for the common garden test. At 227 dph, the second phase of nutritional challenge started by feeding either low HUFA diet LH2 or high-starch/low-protein diet HG2 in each of the two tanks where two groups cohabited, namely, groups C1-LH2 and LH1-LH2 on the one hand, and groups C1-HG2 and HG1-HG2 on the other hand. HUFA were restricted in diet LH2 by replacing 65 % of the protein sources by lupin meal (c. 0.5 % HUFA, dry matter basis), while 30 % of the protein sources were replaced by starch in diet HG2. The fish were fed these experimental diets for two months, until the end of the experiment. They were individually weighed at 225, 266 and 287 dph after light anaesthesia (2-phenoxyethanol, 200 μL L^−1^). The individual specific growth rate (SGR) was computed between the last two weighing times (266 and 287 dph).

### Final hypoxic challenge test

At 292 dph, the fish were grouped in one tank, and fasted for one day before being challenged for resistance to hypoxia. The method of this challenge test was previously described [41, 42]. Briefly, the rearing water was deprived of oxygen by bubbling nitrogen gas in the tank, under constant monitoring of dissolved O_2_. The oxygenation level was first dropped by 90 % from saturation within 1 h, and then further decreased by 1.2 % per hour, until reaching a minimum of c. 4 % air saturation. In the meantime, the fish were constantly observed, and each individual losing its maintenance of equilibrium was identified by reading its PIT-tag, and immediately placed in a fully aerated tank for recovery. The challenge lasted c. 8 h in total, and the interval of time from start to equilibrium lost was noted for every fish as its hypoxia resistance time (HRT).

### Sampling for microbiological analysis

In each group, the fish were sorted based on HRT, and 12 individuals were selected per group for microbiological sampling (60 fish in total). Four individuals were selected among the most sensitive to hypoxia in each group, four others were among the mildly sensitive, and the four last were among the most resistant. As there were significant differences in hypoxia sensitivity between groups, the selection grid was not the same in every group [see Additional file 10].

At 294 dph, after two days of fasting, the 60 selected fish were euthanized with an overdose of anaesthetic (2-phenoxyethanol, 1 mL L^−1^). The intestines were empty, and each one was dissected under sterile conditions, and separated from the perivisceral fat in a Petri dish on ice. The intestine was immediately plunged into a microtube with 1.5 mL RNAlater (Qiagen). After soaking for 24 h at c. 4 °C, the tubes were stored at −80 °C.

### RNA extraction, RT-PCR, and pyrosequencing

The microbial profiles compared in the present experiment were based on the analysis of 16S rRNA after reverse transcription, which was preferred to the method based on genomic DNA. As explained previously [16], this is a way to focus on the relative ribosomal activity among bacteria, whereas the relative abundance of rDNA cannot provide information about bacterial activity, and the result is biased by the variable number of gene copies among species.

After thawing, the intestine was removed from RNAlater with sterile tweezers, cut open longitudinally along the entire length in a Petri dish, and plunged into Extract-All (Eurobio) chilled on ice. RNA was extracted according to the instructions of the manufacturer for biological tissues, with an additional step of bead-beating for 10 min after the initial step of homogenization with a dispersing aggregate unit. After purification, the RNA concentration was estimated by NanoDrop (Thermo Scientific), and aliquoted for reverse transcription (RT). The surplus was precipitated and stored at −80 °C. cDNA was transcribed with the QuantiTect® RT kit (Qiagen), and stored at −20 °C.

Due to the small proportion of 16S rRNA in the samples, nested PCR was required before pyrosequencing. The PCR mix contained Taq DNA polymerase (0.025 U μl^−1^; MP Biomedicals), 0.2 mM of each dNTP (deoxyribonucleotide triphosphate; Eurogentec premix), and 0.4 μM of each primer (first round: EUB-8-f, 907-r; second round, V3-V4 region: PCR1F_460 and PCR1R_460 [see Additional file 12]). After initial denaturation at 94 °C for 2 min in T100 Thermal Cycler (Bio-Rad), 20 and 25 cycles were run in the first and second round, respectively, with 30 s denaturation at 94 °C, 30 s annealing at 55 °C (first round) or 62 °C (second round), and 1 min elongation at 72 °C. Both rounds ended with 1 min extension at 72 °C. Six of the sixty samples were ruled out because of insufficient PCR yield [see Additional file 10]. The 54 other PCR products were purified with GenElute PCR Clean-Up Kit (Sigma), further prepared by GenPhySE (INRA, UMR1388, Toulouse, France), and sequenced with Illumina MiSeq at GeT-PlaGe [43]. The pyrosequencing data were deposited in the National Center for Biotechnology Information-Short Reads Archive (NCBI-SRA) under the BioProject accession number PRJNA294963 [44].

### Bioinformatics data processing

The raw sequence dataset was first treated with FROGS (Find Rapidly OTU with Galaxy Solution) [45]. Briefly, after merging the paired 250 bp reads, the software denoised the dataset, which was clustered with Swarm [46]. A first range of chimera was removed with vsearch [47], and then the dataset was further filtered using PhiX and removing the singletons. A second filtration level was obtained by keeping only the clusters that represented at least 0.0002 % of total reads. After double identification with RDP and Blast + the dataset was restricted to the bacterial kingdom. A total of 1160 different sequences were thus detected among the 1,017,693 remaining reads. Between 11,599 and 28,067 valid reads were counted in each sample, and the data were normalized on the basis of 11,599 reads per sample, before computing alpha-diversity and the rarefaction curves. The normalization resulted in 1111 operational taxonomic units (OTUs). The final identification was assigned at the lowest phylogenetic level of RDP-Blast concordance, after correcting some misleading affiliations.

### Statistics

Fish growth and resistance to hypoxia, the diversity indices of intestinal microbiota, and the relative abundance of OTUs were compared between experimental groups by ANOVA or Kruskal-Wallis test, depending on normality and homoscedasticity. Post-hoc tests were used for multiple comparisons between dietary groups (Tukey’s and Dunn’s tests after ANOVA and Kruskal-Wallis, respectively). It must be noticed that the most relevant pairwise comparisons in the present experiment were those between the groups (1) with or without the deficient diet at first feeding and reared in the same tank during the final phase (LH1-LH2 vs. C1-LH2; HG1-HG2 vs. C1-HG2) and (2) with standard diet at first feeding, but either transferred or not from the control tank before the final phase (C1-LH2, C1-HG2, and C1-C2). The Bray-Curtis index was used for comparing the similarity between bacterial profiles by PERMANOVA with PAST [48]. The bacterial profiles were further compared between two groups by Linear Discriminant Analysis (LDA) Effective Size (LEfSe) pairwise analysis under Galaxy environment [49, 50]. The canonical correlation between intestinal microbiota and fish growth or resistance to hypoxia was analysed after (sparse) Partial Least Squares, (s)PLS, classification with mixOmics [51, 52].

## Additional files

Additional file 1: Rarefaction curves of OTU richness of each sample from the experimental groups, computed after normalization to 11599 reads per sample. (PPTX 916 kb)
Additional file 2: Venn diagram showing the distribution of the 1111 OTUs among the intestinal samples of the five experimental groups. (PPTX 233 kb)
Additional file 3: Histogram showing the overwhelming dominance of Alpha- and Gamma-Proteobacteria among the OTUs detected in every experimental group. (PPTX 65 kb)
Additional file 4: Histogram showing the distribution of the most dominant OTUs (more than 5 % total reads in at least one sample). (PPTX 2597 kb)
Additional file 5: Mean relative abundance of phylogenetic clusters among Gammaproteobacteria with significant differences between experimental groups. (DOCX 21 kb)
Additional file 6: Mean relative abundance of phylogenetic clusters among Alpha- and Beta-Proteobacteria with significant differences between experimental groups. (DOCX 19 kb)
Additional file 7: Mean relative abundance of phylogenetic clusters among Actinobacteria and Firmicutes with significant differences between experimental groups. (DOCX 19 kb)
Additional file 8: Mean relative abundance of phylogenetic clusters among Bacteroidetes and Spirochaetae with significant differences between experimental groups. (DOCX 18 kb)
Additional file 9: Mean weights (± SE) of sea bass before, at midterm, and after the final dietary challenge of sea bass. (DOCX 15 kb)
Additional file 10: Hypoxia resistance time (h) of the individuals selected for the microbiological analysis. (DOCX 18 kb)
Additional file 11: Phylogenetic tree derived by neighbour joining of the three *Bacillus* sp. OTUs from the present intestinal samples, aligned with GenBank sequences. (PPTX 64 kb)
Additional file 12: Sequences of the primers used in the nested PCR for pyrosequencing. (DOCX 15 kb)

## Abbreviations

ANOVA: Analysis Of VAriance
Blast: Basic local alignment search tool
C: Control diet (C1 and C2, also used to name experimental groups)
cDNA: complementary DNA
DGGE: Denaturing Gradient Gel Electrophoresis
DNA: Deoxyribo-Nucleic Acid
dph: Day post hatch
FROGS: Find Rapidly OTU with Galaxy Solution
HG: Hyper-Glucidic diet (HG1 and HG2, also used to name experimental groups)
HRT: Hypoxia Resistance Time
HUFA: Highly poly-Unsaturated Fatty Acids
LDA: Linear Discriminant Analysis
LEfSe: Linear discriminant Effective Size
LH: Low-HUFA diet (LH1 and LH2, also used to name experimental groups)
OTU: Operational Taxonomy Unit
PAST: PAleontological STatistics software package
PCR: Polymerase Chain Reaction
PERMANOVA: PERmutational Multivariate Analysis Of Variance
PLS: Partial Least Squares
RDP: Ribosomal Database Project
rRNA: ribosomal Ribo-Nucleic Acid
RT-PCR: Reverse Transcription Polymerase Chain Reaction
SE: Standard error
SGR: Specific Growth Rate
sPLS: sparse Partial Least Squares
