# Supplementary material for: The highly variable microbiota associated to intestinal mucosa correlates with growth and hypoxia resistance of sea bass, Dicentrarchus labrax, submitted to different nutritional histories
Source: BMC Microbiol. 2016 Nov 8;16:266. doi: 10.1186/s12866-016-0885-2 (PMC5100225; doi:10.1186/s12866-016-0885-2)
Supplement: Additional file 1: — Rarefaction curves of OTU richness of each sample from the experimental groups, computed after normalization to 11599 reads per sample. (PPTX 916 kb) [file 12866_2016_885_MOESM1_ESM.pptx]

## Slide 1
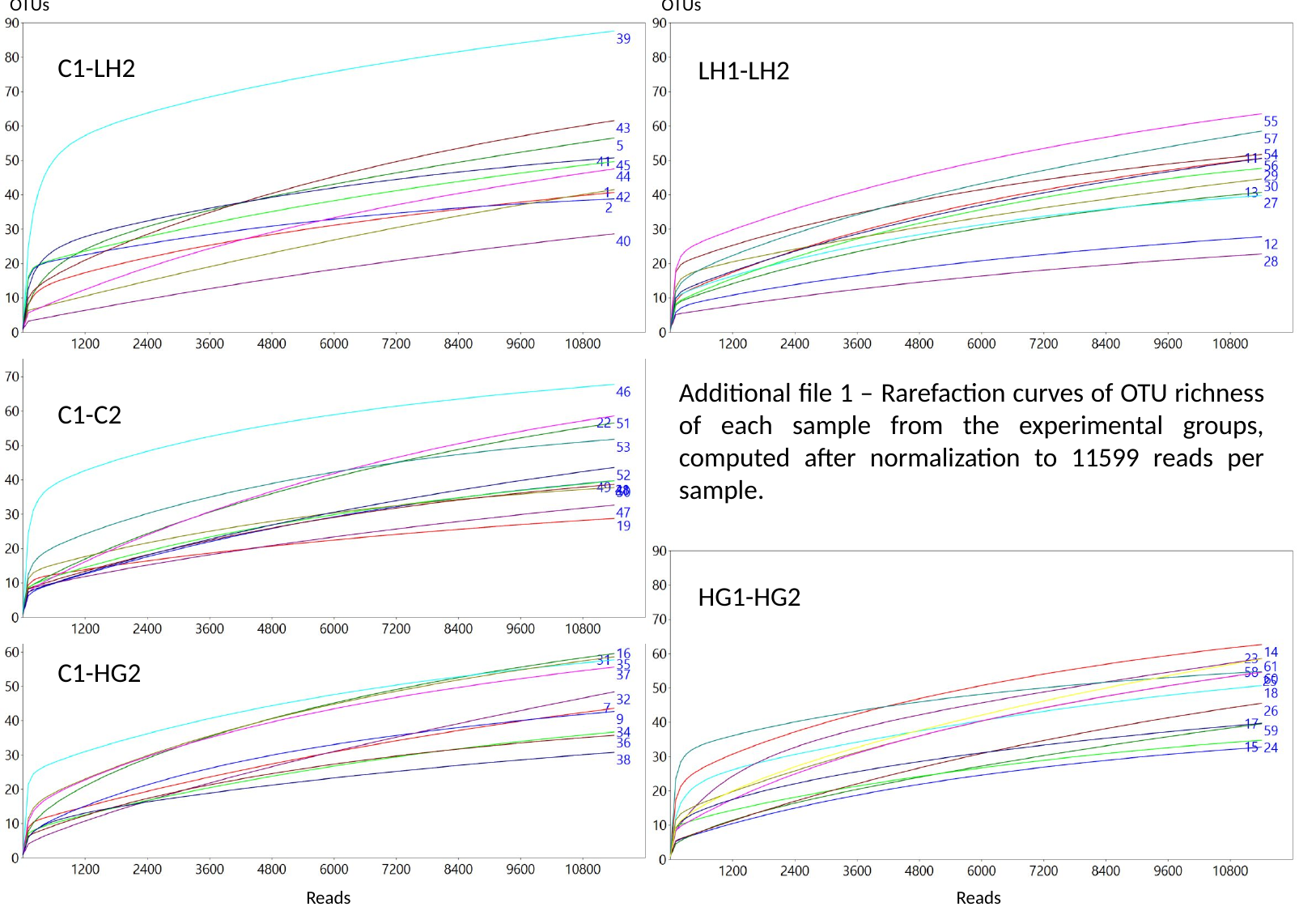

OTUs
OTUs
C1-LH2
LH1-LH2
Additional file 1 – Rarefaction curves of OTU richness of each sample from the experimental groups, computed after normalization to 11599 reads per sample.
C1-C2
HG1-HG2
C1-HG2
Reads
Reads
