# Supplementary material for: The highly variable microbiota associated to intestinal mucosa correlates with growth and hypoxia resistance of sea bass, Dicentrarchus labrax, submitted to different nutritional histories
Source: BMC Microbiol. 2016 Nov 8;16:266. doi: 10.1186/s12866-016-0885-2 (PMC5100225; doi:10.1186/s12866-016-0885-2)
Supplement: Additional file 2: — Venn diagram showing the distribution of the 1111 OTUs among the intestinal samples of the five experimental groups. (PPTX 233 kb) [file 12866_2016_885_MOESM2_ESM.pptx]

## Slide 1
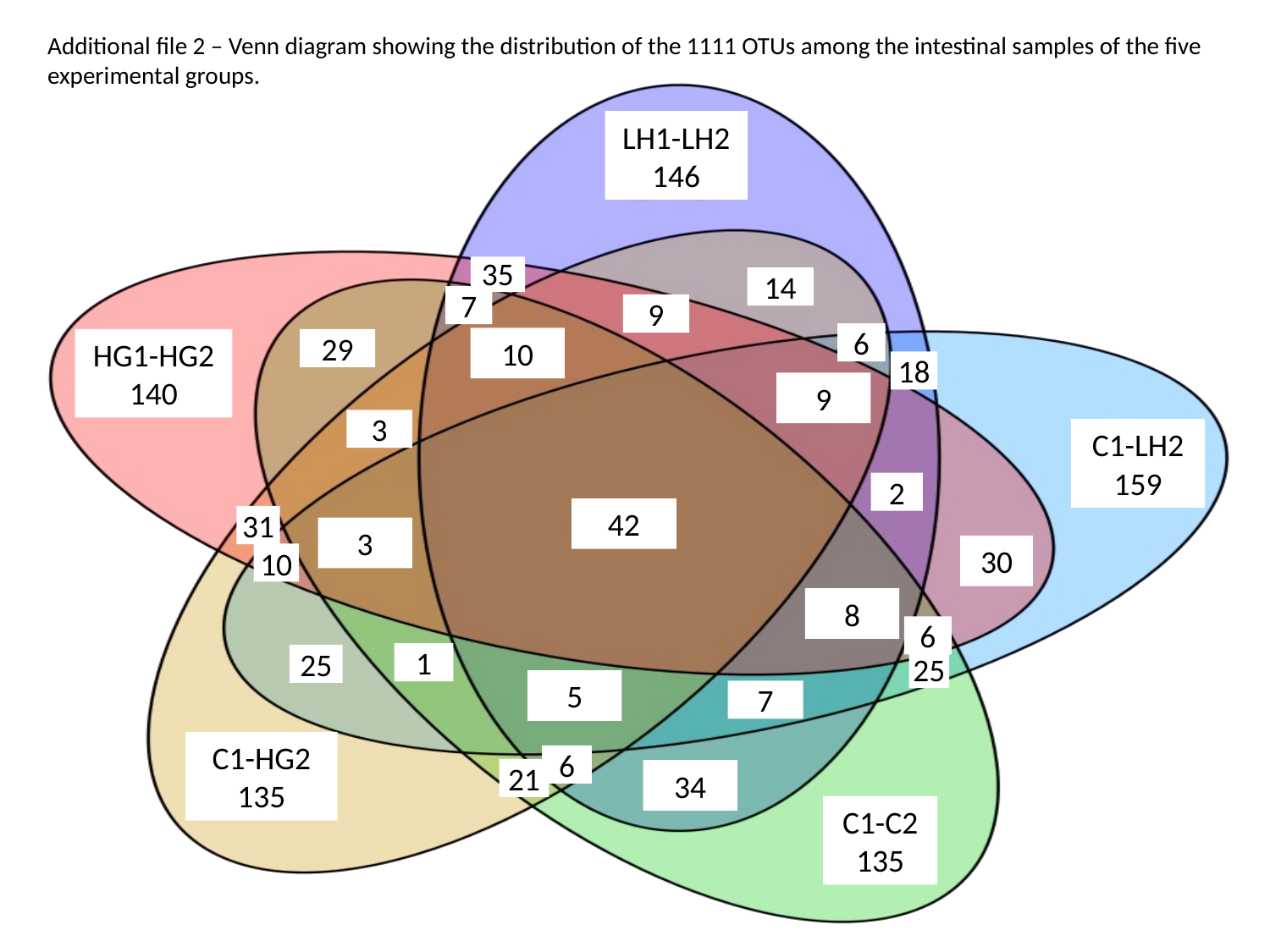

Additional file 2 – Venn diagram showing the distribution of the 1111 OTUs among the intestinal samples of the five experimental groups.
LH1-LH2
146
35
14
7
9
6
10
HG1-HG2
140
29
18
9
3
C1-LH2
159
2
42
31
3
30
10
8
6
1
25
25
5
7
C1-HG2
135
6
21
34
C1-C2
135
