# Supplementary material for: The highly variable microbiota associated to intestinal mucosa correlates with growth and hypoxia resistance of sea bass, Dicentrarchus labrax, submitted to different nutritional histories
Source: BMC Microbiol. 2016 Nov 8;16:266. doi: 10.1186/s12866-016-0885-2 (PMC5100225; doi:10.1186/s12866-016-0885-2)
Supplement: Additional file 3: — Histogram showing the overwhelming dominance of Alpha- and Gamma-Proteobacteria among the OTUs detected in every experimental group. (PPTX 65 kb) [file 12866_2016_885_MOESM3_ESM.pptx]

## Slide 1
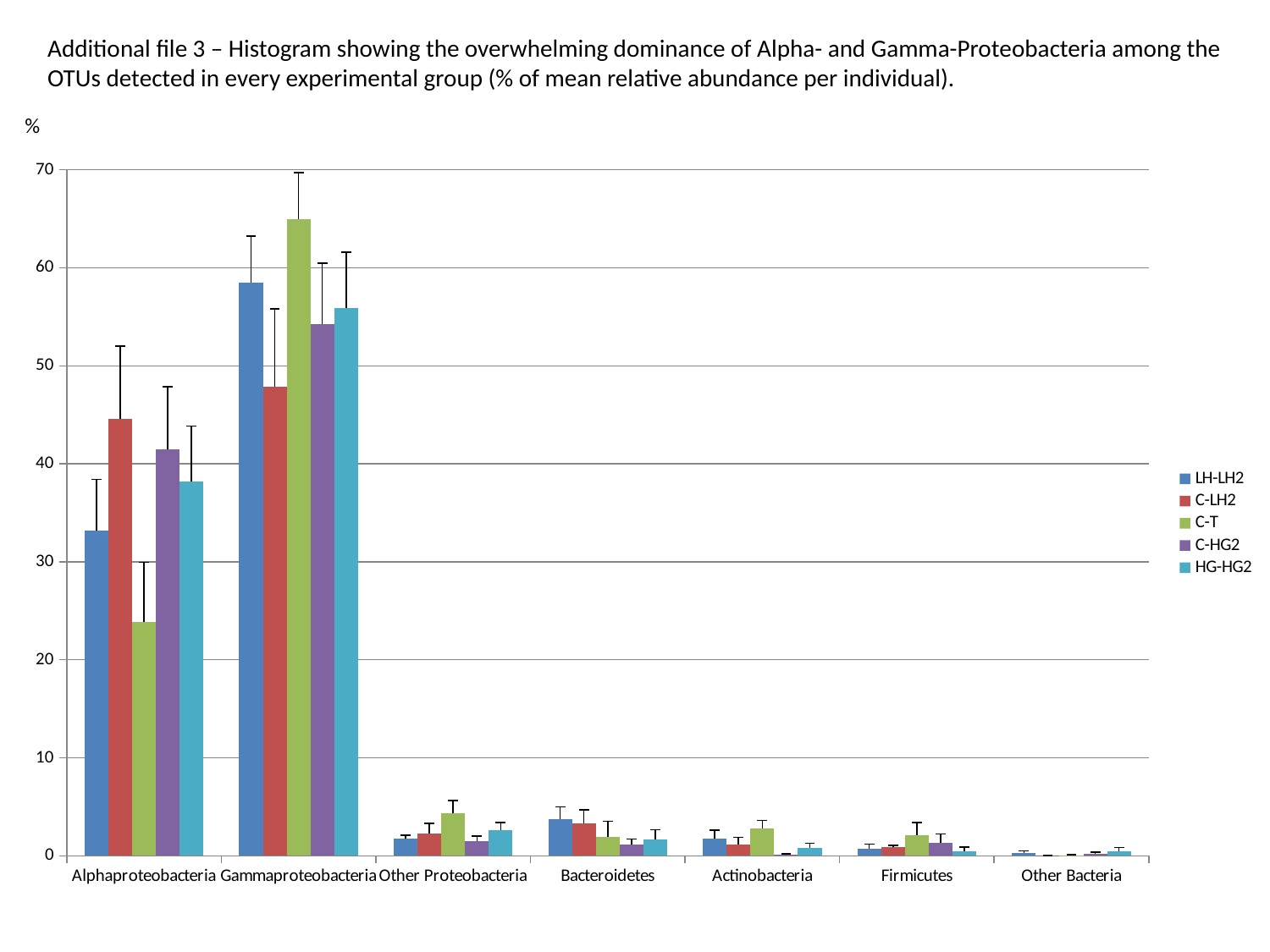

Additional file 3 – Histogram showing the overwhelming dominance of Alpha- and Gamma-Proteobacteria among the
OTUs detected in every experimental group (% of mean relative abundance per individual).
%
### Chart
| Category | LH-LH2 | C-LH2 | C-T | C-HG2 | HG-HG2 |
|---|---|---|---|---|---|
| Alphaproteobacteria | 33.22067496724354 | 44.56592228542248 | 23.856817350161535 | 41.5094166117527 | 38.1620092692759 |
| Gammaproteobacteria | 58.52318514386557 | 47.829928608443225 | 64.97116707272868 | 54.23211435796867 | 55.882026734340464 |
| Other Proteobacteria | 1.7325454781223113 | 2.2966371205692586 | 4.298929203831965 | 1.4682789538316237 | 2.5775838641683246 |
| Bacteroidetes | 3.736557211376373 | 3.2752535176211217 | 1.903285748471933 | 1.1307734600790877 | 1.6325067169856016 |
| Actinobacteria | 1.7732167933509573 | 1.1772613497208668 | 2.828538463267251 | 0.11046768595316872 | 0.8234782449714686 |
| Firmicutes | 0.7523724125630996 | 0.8532652103459698 | 2.077065681011752 | 1.335595493591969 | 0.46090132376096865 |
| Other Bacteria | 0.25648021191425685 | 0.0004831384674847812 | 0.06209234282620264 | 0.2099160588234777 | 0.45709625859711006 |
