# Supplementary material for: The highly variable microbiota associated to intestinal mucosa correlates with growth and hypoxia resistance of sea bass, Dicentrarchus labrax, submitted to different nutritional histories
Source: BMC Microbiol. 2016 Nov 8;16:266. doi: 10.1186/s12866-016-0885-2 (PMC5100225; doi:10.1186/s12866-016-0885-2)
Supplement: Additional file 4: — Histogram showing the distribution of the most dominant OTUs (more than 5 % total reads in at least one sample). (PPTX 2597 kb) [file 12866_2016_885_MOESM4_ESM.pptx]

## Slide 1
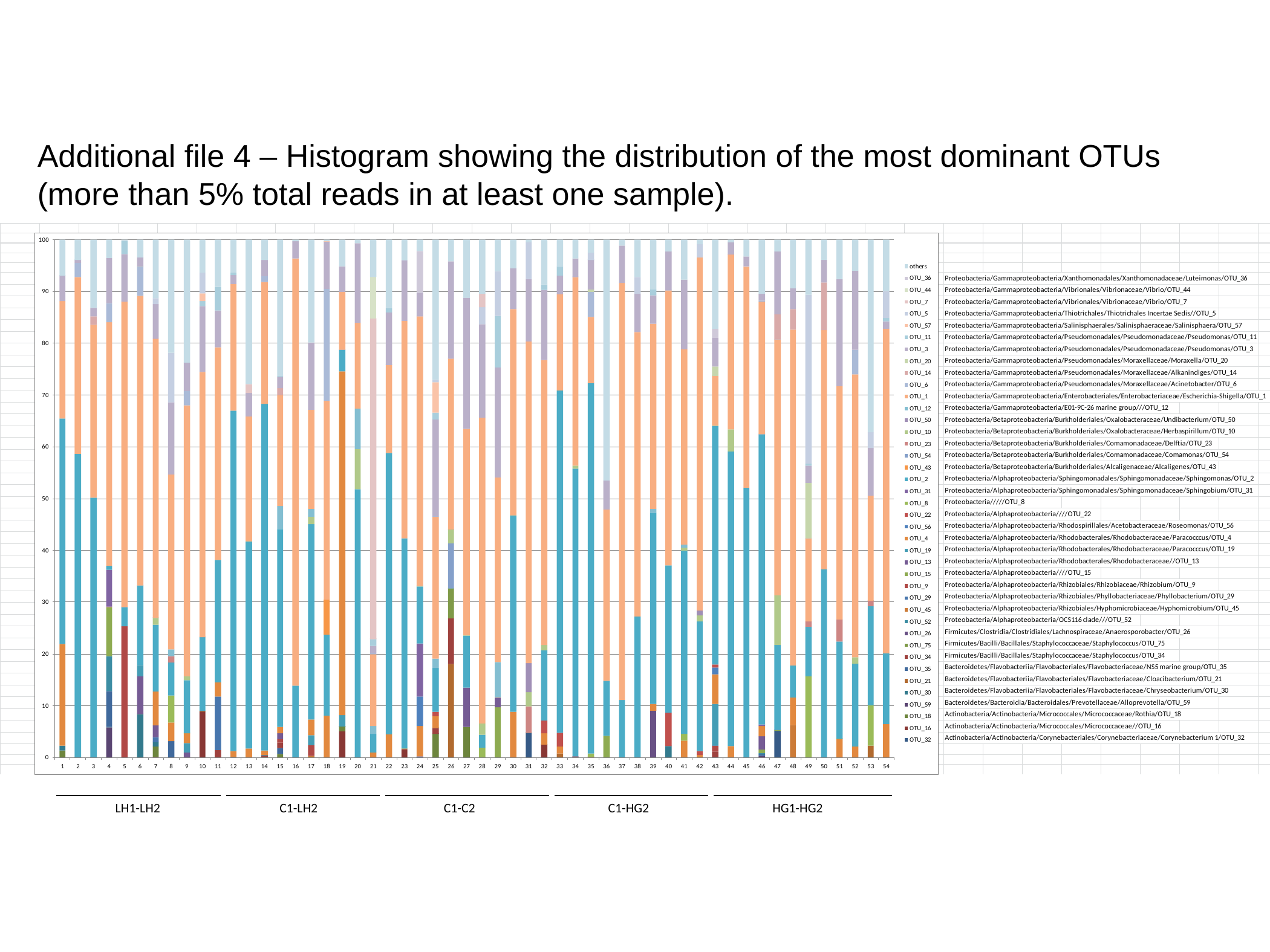

Additional file 4 – Histogram showing the distribution of the most dominant OTUs (more than 5% total reads in at least one sample).
LH1-LH2
C1-LH2
C1-C2
C1-HG2
HG1-HG2
