# Supplementary material for: The highly variable microbiota associated to intestinal mucosa correlates with growth and hypoxia resistance of sea bass, Dicentrarchus labrax, submitted to different nutritional histories
Source: BMC Microbiol. 2016 Nov 8;16:266. doi: 10.1186/s12866-016-0885-2 (PMC5100225; doi:10.1186/s12866-016-0885-2)
Supplement: Additional file 5: — Mean relative abundance of phylogenetic clusters among Gammaproteobacteria with significant differences between experimental groups. (DOCX 21 kb) [file 12866_2016_885_MOESM5_ESM.docx]

**Additional file 5 Mean relative abundance of phylogenetic clusters among Gammaproteobacteria with significant differences between experimental groups.**

| Class / Order / Family / *Genus* / OTU | LH1-LH2 | C1-LH2 | C1-C2 | C1-HG2 | HG1-HG2 | Test | *P** |
| --- | --- | --- | --- | --- | --- | --- | --- |
| Proteobacteria / Gammaproteobacteria / | 58.6^yz^ ± 3.5 | 47.8^yz^ ± 6.5 | 65.0^y^ ± 4.7 | 54.1^z^ ± 6.3 | 55.9^yz^ ± 5.7 | LEfSe | ≤0.001 |
| Gammaproteobacteria / Enterobacteriales / Enterobacteriaceae / *Escherichia*-*Shigella* / OTU_1 | 44.0^yz^ ± 3.5 | 27.5^z^ ± 6.5 | 42.1^y^ ± 4.2 | 43.1^yz^ ± 6.7 | 39.2^y^ ± 5.2 | LEfSe | 0.009 |
| Gammaproteobacteria / Enterobacteriales / Enterobacteriaceae / *Escherichia*-*Shigella* / OTU_220 | 0.0133^yz^ ± 0.0051 | 0.0043^z^ ± 0.0035 | 0.0227^y^ ± 0.0069 | 0.0078^yz^ ± 0.0020 | 0.0165^yz^ ± 0.0048 | LEfSe | 0.007 |
| Gammaproteobacteria / Enterobacteriales / Enterobacteriaceae / *Escherichia*-*Shigella* / OTU_223 | 0.0039^z^ ± 0.0021 | 0.0198^y^ ± 0.0082 | 0.0039^z^ ± 0.0024 | 0.0181^y^ ± 0.0047 | 0.0115^yz^ ± 0.0042 | KW | 0.019 |
| Gammaproteobacteria / Pseudomonadales | 10.7^ab^ ± 1.5 | 9.3^ab^ ± 2.8 | 17.0^a^ ± 2.3 | 7.2^b^ ± 1.3 | 11.0^ab^ ± 2.0 | ANOVA | 0.021 |
| Gammaproteobacteria / Pseudomonadales / Pseudomonadaceae / *Pseudomonas* | 8.3^b^ ± 1.6 | 6.9^b^ ± 1.5 | 16.2^a^ ± 2.3 | 6.7^b^ ± 1.2 | 7.2^b^ ± 1.8 | ANOVA | ≤0.001 |
| Gammaproteobacteria / Pseudomonadales / Pseudomonadaceae / *Pseudomonas* / OTU_3 | 6.6^b^ ± 1.3 | 5.9^b^ ± 1.6 | 14.7^a^ ± 1.9 | 6.0^b^ ± 1.1 | 6.8^b^ ± 1.8 | ANOVA | ≤0.001 |
| Gammaproteobacteria / Pseudomonadales / Pseudomonadaceae / *Pseudomonas* / OTU_291 | 0.0008^z^ ± 0.0008 | 0.0121^y^ ± 0.0050 | 0.0016^z^ ± 0.0016 | 0.0060^yz^ ± 0.0029 | 0.0036^yz^ ± 0.0020 | LEfSe | 0.034 |
| Gammaproteobacteria / Pseudomonadales / Moraxellaceae | 2.45^ab^ ± 0.64 | 2.49^ab^ ± 2.14 | 0.77^b^ ± 0.61 | 0.55^b^ ± 0.54 | 3.81^a^ ± 1.08 | KW | 0.002 |
| Gammaproteobacteria / Pseudomonadales / Moraxellaceae / *Acinetobacter* | 2.21^y^ ± 0.67 | 2.35^yz^ ± 2.15 | 0.61^yz^ ± 0.46 | 0.51^z^ ± 0.50 | 1.23^yz^ ± 0.58 | LEfSe | 0.040 |
| Gammaproteobacteria / Pseudomonadales / Moraxellaceae / *Alkanindiges* / OTU_14 | 0.144^ab^ ± 0.143 | 0.139^ab^ ± 0.139 | 0.002^ab^ ± 0.002 | 0^b^ | 1.526^a^ ± 0.860 | KW | 0.034 |
| Gammaproteobacteria / Thiotrichales / OTU_5 | 1.344^yz^ ± 0.904 | 0.002^z^ ± 0.001 | 1.787^y^ ± 0.961 | 0.716^yz^ ± 0.389 | 3.402^yz^ ± 2.701 | LEfSe | 0.015 |
| Gammaproteobacteria / E01-9C-26 marine group | 0.17^y^ ± 0.13 | 1.58^y^ ± 0.85 | 0.80^yz^ ± 0.64 | 0.24^yz^ ± 0.18 | 0^z^ | LEfSe | 0.024 |
| Gammaproteobacteria / E01-9C-26 marine group / OTU_12 | 0.12^yz^ ± 0.12 | 1.51^y^ ± 0.83 | 0.80^yz^ ± 0.63 | 0.14^yz^ ± 0.09 | 0^z^ | LEfSe | 0.019 |
| Gammaproteobacteria / E01-9C-26 marine group / OTU_540 | 0^z^ | 0.0060^y^ ± 0.0034 | 0.0008^yz^ ± 0.0008 | 0^z^ | 0^z^ | KW | 0.038 |
| Gammaproteobacteria / Salinisphaerales / Salinisphaeraceae / *Salinisphaera* | 0.538^yz^ ± 0.407 | 0.060^yz^ ± 0.060 | 0.324^y^ ± 0.318 | 0^z^ | 0.344^yz^ ± 0.262 | LEfSe | 0.018 |
| Gammaproteobacteria / Oceanospirillales / SAR86 clade / OTU_67 | 0.0008^yz^ ± 0.0008 | 0^z^ | 0^z^ | 0.420^y^ ± 0.411 | 0.237^yz^ ± 0.196 | KW | 0.027 |
| Gammaproteobacteria / Xanthomonadales / JTB255 marine benthic group | 0^b^ | 0.29^a^ ± 0.17 | 0^b^ | 0^b^ | 0^b^ | KW | 0.008 |

The differences were compared between all groups, simultaneously (ANOVA or KW: Kruskal-Wallis test), and between pairs after Linear Discriminant Analysis (LDA) Effective Size (LEfSe) comparisons. The mean percentages with a single superscript a or b on the same line corresponded to the significant differences according to the post-hoc pairwise comparisons (Tukey’s and Dunn’s methods for ANOVA and KW, respectively). The means with a single superscript y or z on the same line corresponded to significant differences after LEfSe pairwise comparisons (not shown in case of significant difference with ANOVA or KW on the 3 groups); *in case of LEfSe , only the lowest *p* among the multiple pairwise comparisons was shown.
