# Supplementary material for: The highly variable microbiota associated to intestinal mucosa correlates with growth and hypoxia resistance of sea bass, Dicentrarchus labrax, submitted to different nutritional histories
Source: BMC Microbiol. 2016 Nov 8;16:266. doi: 10.1186/s12866-016-0885-2 (PMC5100225; doi:10.1186/s12866-016-0885-2)
Supplement: Additional file 6: — Mean relative abundance of phylogenetic clusters among Alpha- and Beta-Proteobacteria with significant differences between experimental groups. (DOCX 19 kb) [file 12866_2016_885_MOESM6_ESM.docx]

**Additional file 6 Mean relative abundance of phylogenetic clusters among Alpha- and Beta-Proteobacteria with significant differences between experimental groups.**

| Class / Order / Family / *Genus* / OTU | LH1-LH2 | C1-LH2 | C1-C2 | C1-HG2 | HG1-HG2 | Test | *P** |
| --- | --- | --- | --- | --- | --- | --- | --- |
| Proteobacteria / Alphaproteobacteria / | 32.7^yz^ ± 5.4 | 44.6^y^ ± 7.5 | 23.1^z^ ± 6.2 | 40.1^yz^ ± 6.4 | 35.9^yz^ ± 6.0 | LEfSe | 0.035 |
| Alphaproteobacteria / Sphingomonadales | 22.8^yz^ ± 5.8 | 34.0^yz^ ± 7.5 | 17.3^z^ ± 5.8 | 37.2^y^ ± 6.7 | 29.8^yz^ ± 5.7 | LEfSe | 0.024 |
| Alphaproteobacteria / Sphingomonadales / Sphingomonadaceae / *Sphingomonas* / OTU_2 | 21.8^yz^ ± 6.0 | 33.8^yz^ ± 7.5 | 16.2^z^ ± 5.7 | 36.8^yz^ ± 6.7 | 28.9^y^ ± 5.4 | LEfSe | 0.042 |
| Alphaproteobacteria / Sphingomonadales / Sphingomonadaceae / *Sphingomonas* / OTU_169 | 0.0149^yz^ ± 0.0042 | 0.0250^yz^ ± 0.0078 | 0.0102^z^ ± 0.0040 | 0.0362^yz^ ± 0.0131 | 0.0374^y^ ± 0.0103 | LEfSe | 0.048 |
| Alphaproteobacteria / Sphingomonadales / Sphingomonadaceae / *Sphingomonas* / OTU_201 | 0.0063^z^ ± 0.0020 | 0.0190^yz^ ± 0.0051 | 0.0149^yz^ ± 0.0067 | 0.0276^y^ ± 0.0073 | 0.0381^yz^ ± 0.0114 | LEfSe | 0.026 |
| Alphaproteobacteria / Sphingomonadales / Sphingomonadaceae / *Sphingomonas* / OTU_271 | 0.0008^z^ ± 0.0008 | 0.0035^yz^ ± 0.0014 | 0.0031^yz^ ± 0.0021 | 0.0069^yz^ ± 0.0042 | 0.0065^y^ ± 0.0022 | LEfSe | 0.028 |
| Alphaproteobacteria / Sphingomonadales / Sphingomonadaceae / *Sphingobium* / OTU_31 | 0.643^yz^ ± 0.641 | 0^z^ | 0.932^yz^ ± 0.932 | 0.007^yz^ ± 0.007 | 0.028^y^ ± 0.026 | LEfSe | 0.0499 |
| Alphaproteobacteria / Rhodospirillales / Rhodospirillaceae | 0^z^ | 0.18^yz^ ± 0.12 | 0^z^ | 0.23^yz^ ± 0.23 | 0.31^y^ ± 0.22 | LEfSe | 0.040 |
| Betaproteobacteria | 1.36^yz^ ± 0.37 | 2.15^yz^ ± 1.01 | 3.77^y^ ± 1.37 | 1.09^z^ ± 0.38 | 2.41^yz^ ± 0.85 | LEfSe | 0.049 |
| Betaproteobacteria / Burkholderiales / Comamonadaceae | 0.50^ab^ ± 0.29 | 0.08^b^ ± 0.05 | 2.31^a^ ± 0.87 | 0.16^b^ ± 0.15 | 0.78^ab^ ± 0.50 | KW | 0.042 |
| Betaproteobacteria / Burkholderiales / Oxalobacteraceae / *Herbaspirillum* / OTU_10 | 0.19^z^ ± 0.13 | 0.93^yz^ ± 0.77 | 0.78^y^ ± 0.35 | 0.22^yz^ ± 0.12 | 1.26^yz^ ± 0.83 | LEfSe | 0.043 |
| Betaproteobacteria / Burkholderiales / Oxalobacteraceae / *Undibacterium* / OTU_50 | 0.0008^yz^ ± 0.0008 | 0^z^ | 0.513^y^ ± 0.509 | 0.0862^yz^ ± 0.0862 | 0.0014^yz^ ± 0.0010 | LEfSe | 0.040 |

The differences were compared between all groups, simultaneously (KW: Kruskal-Wallis test), and between pairs after Linear Discriminant Analysis (LDA) Effective Size (LEfSe) comparisons. The mean percentages with a single superscript a or b on the same line corresponded to the significant differences according to the post-hoc pairwise comparisons (Dunn’s method). The means with a single superscript y or z on the same line corresponded to significant differences after LEfSe pairwise comparisons (not shown in case of significant difference with KW on the 3 groups); *in case of LEfSe , only the lowest *p* among the multiple pairwise comparisons was shown.
