# Supplementary material for: The highly variable microbiota associated to intestinal mucosa correlates with growth and hypoxia resistance of sea bass, Dicentrarchus labrax, submitted to different nutritional histories
Source: BMC Microbiol. 2016 Nov 8;16:266. doi: 10.1186/s12866-016-0885-2 (PMC5100225; doi:10.1186/s12866-016-0885-2)
Supplement: Additional file 7: — Mean relative abundance of phylogenetic clusters among Actinobacteria and Firmicutes with significant differences between experimental groups. (DOCX 19 kb) [file 12866_2016_885_MOESM7_ESM.docx]

**Additional file 7 Mean relative abundance of phylogenetic clusters among Actinobacteria and Firmicutes with significant differences between experimental groups.**

| Phylum / Class / Order / Family / *Genus* / OTU | LH1-LH2 | C1-LH2 | C1-C2 | C1-HG2 | HG1-HG2 | Test | *P** |
| --- | --- | --- | --- | --- | --- | --- | --- |
| Actinobacteria (phylum) | 1.77^ab^ ± 0.83 | 1.20^ab^ ± 0.73 | 2.88^a^ ± 0.80 | 0.11^b^ ± 0.09 | 0.84^ab^ ± 0.47 | KW | 0.031 |
| Actinobacteria / Actinobacteria (class) | 1.77^ab^ ± 0.83 | 1.14^ab^ ± 0.71 | 2.82^a^ ± 0.77 | 0.11^b^ ± 0.09 | 0.82^ab^ ± 0.47 | KW | 0.029 |
| Actinobacteria / Actinobacteria / Micrococcales | 1.69^y^ ± 0.83 | 0.92^y^ ± 0.71 | 1.82^y^ ± 0.69 | 0.09^z^ ± 0.09 | 0.22^yz^ ± 0.10 | KW | 0.049 |
| Actinobacteria / Actinobacteria / Micrococcales / Micrococcaceae | 1.29^yz^ ± 0.84 | 0.89^y^ ± 0.70 | 1.58^y^ ± 0.72 | 0.09^z^ ± 0.09 | 0.08^yz^ ± 0.06 | LEfSe | 0.032 |
| Actinobacteria / Actinobacteria / Micrococcales / Micrococcaceae / *Rothia* / OTU_18 | 0.307^yz^ ± 0.210 | 0.176^y^ ± 0.110 | 0.950^yz^ ± 0.643 | 0^z^ | 0^z^ | LEfSe | 0.019 |
| Actinobacteria / Actinobacteria / Corynebacteriales | 0^z^ | 0.20^yz^ ± 0.14 | 1.00^y^ ± 0.53 | 0.02^yz^ ± 0.02 | 0.54^yz^ ± 0.44 | LEfSe | 0.014 |
| Actinobacteria / Actinobacteria / Corynebacteriales / Corynebacteriaceae / *Corynebacterium* | 0^z^ | 0.14^yz^ ± 0.14 | 0.92^y^ ± 0.53 | 0.02^yz^ ± 0.02 | 0.44^yz^ ± 0.43 | LEfSe | 0.032 |
| Firmicutes (phylum) | 0.74^yz^ ± 0.43 | 0.88^y^ ± 0.23 | 2.08^yz^ ± 1.32 | 1.37^yz^ ± 0.92 | 0.44^z^ ± 0.42 | LefSe | 0.030 |
| Firmicutes / Bacilli | 0.53^yz^ ± 0.28 | 0.78^y^ ± 0.24 | 2.07^yz^ ± 1.32 | 0.14^z^ ± 0.08 | 0.20^yz^ ± 0.20 | LefSe | 0.029 |
| Firmicutes / Bacilli / Bacillales | 0.37^yz^ ± 0.23 | 0.68^y^ ± 0.20 | 1.75^y^ ± 1.31 | 0.13^z^ ± 0.08 | 0.19^z^ ± 0.19 | LESe | 0.001 |
| Firmicutes / Bacilli / Bacillales / Staphylococcaceae / *Staphylococcus* | 0.368^yz^ ± 0.233 | 0.394^y^ ± 0.160 | 1.680^yz^ ± 1.317 | 0.0009^z^ ± 0.0009 | 0.194^z^ ± 0.194 | LEfSe | 0.017 |
| Firmicutes / Bacilli / Bacillales / Staphylococcaceae / *Staphylococcus* /OTU_49 | 0.232^yz^ ± 0.204 | 0.253^y^ ± 0.145 | 0.248^yz^ ± 0.168 | 0.0009^yz^ ± 0.0009 | 0^z^ | LEfSe | 0.019 |
| Firmicutes / Bacilli / Bacillales / Bacillaceae | 0^z^ | 0.260^y^ ± 0.140 | 0.002^yz^ ± 0.002 | 0.005^yz^ ± 0.005 | 0^z^ | LEfSe | 0.047 |
| Firmicutes / Bacilli / Bacillales / Bacillaceae / Bacillus | 0^z^ | 0.260^y^ ± 0.140 | 0^z^ | 0.005^yz^ ± 0.005 | 0^z^ | KW | 0.035 |

The differences were compared between all groups, simultaneously (KW: Kruskal-Wallis test), and between pairs after Linear Discriminant Analysis (LDA) Effective Size (LEfSe) comparisons. The mean percentages with a single superscript a or b on the same line corresponded to the significant differences according to the post-hoc pairwise comparisons (Dunn’s method). The means with a single superscript y or z on the same line corresponded to significant differences after LEfSe pairwise comparisons (not shown in case of significant difference with KW on the 3 groups); *in case of LEfSe , only the lowest *p* among the multiple pairwise comparisons was shown.
