# Supplementary material for: The highly variable microbiota associated to intestinal mucosa correlates with growth and hypoxia resistance of sea bass, Dicentrarchus labrax, submitted to different nutritional histories
Source: BMC Microbiol. 2016 Nov 8;16:266. doi: 10.1186/s12866-016-0885-2 (PMC5100225; doi:10.1186/s12866-016-0885-2)
Supplement: Additional file 8: — Mean relative abundance of phylogenetic clusters among Bacteroidetes and Spirochaetae with significant differences between experimental groups. (DOCX 18 kb) [file 12866_2016_885_MOESM8_ESM.docx]

**Additional file 8 Mean relative abundance of phylogenetic clusters among Bacteroidetes and Spirochaetae with significant differences between experimental groups.**

| Phylum / Class / Order / Family / *Genus* / OTU | LH1-LH2 | C1-LH2 | C1-C2 | C1-HG2 | HG1-HG2 | Test | *P** |
| --- | --- | --- | --- | --- | --- | --- | --- |
| Bacteroidetes (phylum) | 3.69^y^ ± 1.24 | 3.22^y^ ± 1.40 | 1.89^z^ ± 1.63 | 1.09^yz^ ± 0.56 | 1.63^yz^ ± 1.02 | LEfSe | 0.006 |
| Bacteroidetes / Flavobacteriia / Flavobacteriales | 2.87^y^ ± 0.86 | 3.21^y^ ± 1.40 | 1.77^z^ ± 1.64 | 1.02^yz^ ± 0.57 | 1.43^y^ ± 1.02 | LEfSe | 0.027 |
| Bacteroidetes / Flavobacteriia / Flavobacteriales / Flavobacteriaceae | 2.86^y^ ± 0.86 | 3.04^yz^ ± 1.35 | 1.77^z^ ± 1.64 | 0.84^yz^ ± 0.42 | 1.28^yz^ ± 0.89 | LEfSe | 0.027 |
| Bacteroidetes / Flavobacteriia / Flavobacteriales / Flavobacteriaceae / *Tenacibaculum* | 0.12^yz^ ± 0.12 | 1.27^y^ ± 1.01 | 0^z^ | 0.16^yz^ ± 0.16 | 0.19^yz^ ± 0.19 | LEfSe | 0.010 |
| Bacteroidetes / Flavobacteriia / Flavobacteriales / Flavobacteriaceae / *Chryseobacterium* / OTU_30 | 0.925^y^ ± 0.754 | 0^z^ | 0.003^yz^ ± 0.003 | 0.222^yz^ ± 0.222 | 0^z^ | KW | 0.046 |
| Bacteroidetes / Flavobacteriia / Flavobacteriales / Flavobacteriaceae / NS5 marine group / OTU_28 | 0.378^yz^ ± 0.378 | 0.463^y^ ± 0.367 | 0^z^ | 0.0009^yz^ ± 0.0009 | 0^z^ | LEfSe | 0.047 |
| Bacteroidetes / Flavobacteriia / Flavobacteriales / Flavobacteriaceae / unknown genus / OTU_38 | 0.0008^yz^ ± 0.0008 | 0.165^yz^ ± 0.165 | 0^z^ | 0.360^yz^ ± 0.342 | 0.300^y^ ± 0.200 | LEfSe | 0.040 |
| Bacteroidetes / Bacteroidia / Bacteroidales /Porphyromonadaceae | 0.0062^yz^ ± 0.0055 | 0.0026^yz^ ± 0.0018 | 0^z^ | 0.0009^yz^ ± 0.0009 | 0.0036^y^ ± 0.0013 | LEfSe | 0.019 |
| Spirochaetae / Spirochaetes / Spirochaetales / Spirochaetaceae / *Treponema* 2 | 0.0016^ab^ ± 0.0011 | 0.0009^ab^ ± 0.0009 | 0.0008^b^ ± 0.0008 | 0.204^ab^ ± 0.167 | 0.445^a^ ± 0.371 | KW | 0.003 |
| Spirochaetae / Spirochaetes / Spirochaetales / Spirochaetaceae / *Treponema* 2 / OTU_47 | 0.0008^b^ ± 0.0008 | 0.0009^ab^ ± 0.0009 | 0^b^ | 0.204^ab^ ± 0.167 | 0.445^a^ ± 0.371 | KW | ≤0.001 |

The differences were compared between all groups, simultaneously (KW: Kruskal-Wallis test), and between pairs after Linear Discriminant Analysis (LDA) Effective Size (LEfSe) comparisons. The mean percentages with a single superscript a or b on the same line corresponded to the significant differences according to the post-hoc pairwise comparisons (Dunn’s method). The means with a single superscript y or z on the same line corresponded to significant differences after LEfSe pairwise comparisons (not shown in case of significant difference with KW on the 3 groups); *in case of LEfSe , only the lowest *p* among the multiple pairwise comparisons was shown.
