# Supplementary material for: The highly variable microbiota associated to intestinal mucosa correlates with growth and hypoxia resistance of sea bass, Dicentrarchus labrax, submitted to different nutritional histories
Source: BMC Microbiol. 2016 Nov 8;16:266. doi: 10.1186/s12866-016-0885-2 (PMC5100225; doi:10.1186/s12866-016-0885-2)
Supplement: Additional file 9: — Mean weights (± SE) of sea bass before, at midterm, and after the final dietary challenge of sea bass. (DOCX 15 kb) [file 12866_2016_885_MOESM9_ESM.docx]

**Additional file 9 Mean weights (± SE) of sea bass before, at midterm, and after the final dietary challenge of sea bass.**

| Age (dph) | 225 | 266 | 287 |
| --- | --- | --- | --- |
| LH1-LH2 | 26.7^ab^ ± 0.5 | 41.9^b^ ± 0.9 | 51.2^c^ ± 1.1 |
| C1-LH2 | 25.8^b^ ± 0.6 | 43.3^b^ ± 1.0 | 54.0^bc^ ± 1.2 |
| C1-C2 | 26.1^b^ ± 0.5 | 43.8^ab^ ± 0.9 | 57.5^ab^ ± 1.1 |
| C1-HG2 | 25.7^b^ ± 0.6 | 42.2^ab^ ± 0.9 | 53.8^abc^ ± 1.1 |
| HG1-HG2 | 28.2^a^± 0.4 | 45.5^a^± 0.8 | 57.9^a^ ± 1.0 |
| *p* (Kruskal-Wallis) | ≤0.001 | 0.007 | ≤0.001 |

SGR was computed from 266 to 287 dph (days post hatch, see Table 3). The means (± SE) without common superscript letter on the same column corresponded to significant differences according to Dunn’s test. Each group was named after its diets during the two challenge phases: HUFA-deficient diet at both phases (LH1-LH2), or only phase 2 (C1-LH2); protein-deficient diet with high starch supply at both phases (HG1-HG2), or only phase 2 (C1-HG2); control with standard diets (C1-C2).
