# Supplementary material for: The highly variable microbiota associated to intestinal mucosa correlates with growth and hypoxia resistance of sea bass, Dicentrarchus labrax, submitted to different nutritional histories
Source: BMC Microbiol. 2016 Nov 8;16:266. doi: 10.1186/s12866-016-0885-2 (PMC5100225; doi:10.1186/s12866-016-0885-2)
Supplement: Additional file 10: — Hypoxia resistance time (h) of the individuals selected for the microbiological analysis. (DOCX 18 kb) [file 12866_2016_885_MOESM10_ESM.docx]

**Additional file 10 Hypoxia resistance time (h) of the individuals selected for the microbiological analysis.**

| Hypoxia sensitivity | Individual | LH-LH2 |  | Individual | C-LH2 |  | Individual | C-T |  | Individual | C-HG2 |  | Individual | HG-HG2 |
| --- | --- | --- | --- | --- | --- | --- | --- | --- | --- | --- | --- | --- | --- | --- |
| Sensitive | LH-LH2-28 | 6.04 |  | C-LH2-42 | 6.16 |  | C-T-47✓ | 6.37 |  | C-HG2-37 | 6.62 |  | HG-HG2-25 | 6.32 |
| Sensitive | LH-LH2-27 | 6.04 |  | C-LH2-44 | 6.18 |  | C-T-46 | 6.37 |  | C-HG2-34 | 6.65 |  | HG-HG2-58 | 6.34 |
| Sensitive | LH-LH2-29✓ | 6.05 |  | C-LH2-5 | 6.20 |  | C-T-50 | 6.37 |  | C-HG2-36 | 6.67 |  | HG-HG2-15 | 6.37 |
| Sensitive | LH-LH2-13 | 6.05 |  | C-LH2-40 | 6.22 |  | C-T-21 | 6.40 |  | C-HG2-31 | 6.68 |  | HG-HG2-59 | 6.39 |
| Mildly sensitive | LH-LH2-55 | 6.52 |  | C-LH2-41 | 6.66 |  | C-T-49 | 6.90 |  | C-HG2-35 | 7.11 |  | HG-HG2-26 | 6.98 |
| Mildly sensitive | LH-LH2-10* | 6.53 |  | C-LH2-39 | 6.69 |  | C-T-20* | 6.92 |  | C-HG2-7 | 7.12 |  | HG-HG2-61 | 7.01 |
| Mildly sensitive | LH-LH2-30✓ | 6.53 |  | C-LH2-1 | 6.69 |  | C-T-22 | 6.98 |  | C-HG2-6* | 7.14 |  | HG-HG2-60 | 7.02 |
| Mildly sensitive | LH-LH2-54 | 6.60 |  | C-LH2-4* | 6.69 |  | C-T-52 | 6.99 |  | C-HG2-16 | 7.17 |  | HG-HG2-14 | 7.03 |
| Resistant | LH-LH2-11 | 7.77 |  | C-LH2-43 | 7.65 |  | C-T-53 | 8.01 |  | C-HG2-32 | 8.17 |  | HG-HG2-17 | 8.00 |
| Resistant | LH-LH2-57 | 7.80 |  | C-LH2-3* | 7.65 |  | C-T-51 | 8.02 |  | C-HG2-38 | 8.19 |  | HG-HG2-24 | 8.00 |
| Resistant | LH-LH2-56 | 7.84 |  | C-LH2-2 | 7.74 |  | C-T-48 | 8.03 |  | C-HG2-33* | 8.22 |  | HG-HG2-23 | 8.00 |
| Resistant | LH-LH2-12 | 7.88 |  | C-LH2-45 | 7.75 |  | C-T-19 | 8.03 |  | C-HG2-9 | 8.26 |  | HG-HG2-18 | 8.02 |

*The quality of the PCR products obtained from the individuals marked in grey was not sufficient for pyrosequencing, and these samples were ruled out from the comparison; ✓fish not weighed at 266 dph, and ruled out from the canonical-correlation analysis.
