# Supplementary material for: The highly variable microbiota associated to intestinal mucosa correlates with growth and hypoxia resistance of sea bass, Dicentrarchus labrax, submitted to different nutritional histories
Source: BMC Microbiol. 2016 Nov 8;16:266. doi: 10.1186/s12866-016-0885-2 (PMC5100225; doi:10.1186/s12866-016-0885-2)
Supplement: Additional file 11: — Phylogenetic tree derived by neighbour joining of the three Bacillus sp. OTUs from the present intestinal samples, aligned with GenBank sequences. (PPTX 64 kb) [file 12866_2016_885_MOESM11_ESM.pptx]

## Slide 1
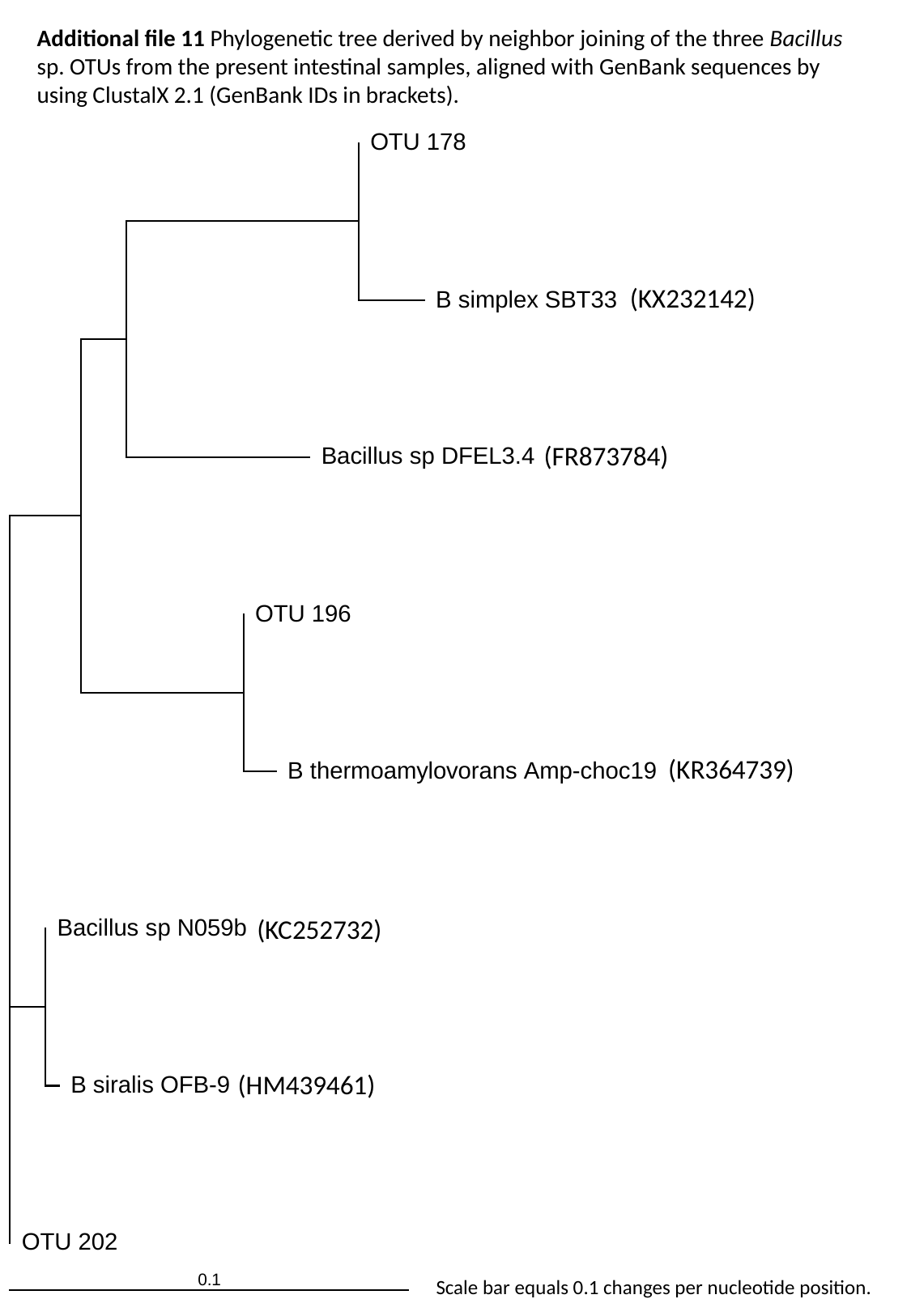

Additional file 11 Phylogenetic tree derived by neighbor joining of the three Bacillus sp. OTUs from the present intestinal samples, aligned with GenBank sequences by using ClustalX 2.1 (GenBank IDs in brackets).
(KX232142)
(FR873784)
(KR364739)
(KC252732)
(HM439461)
 Scale bar equals 0.1 changes per nucleotide position.
