# Supplementary material for: The highly variable microbiota associated to intestinal mucosa correlates with growth and hypoxia resistance of sea bass, Dicentrarchus labrax, submitted to different nutritional histories
Source: BMC Microbiol. 2016 Nov 8;16:266. doi: 10.1186/s12866-016-0885-2 (PMC5100225; doi:10.1186/s12866-016-0885-2)
Supplement: Additional file 12: — Sequences of the primers used in the nested PCR for pyrosequencing. (DOCX 15 kb) [file 12866_2016_885_MOESM12_ESM.docx]

**Additional file 12** Sequences of the primers used in the nested PCR for pyrosequencing.

| Primers (1st round, 16S rRNA) | Sequences | Reference |
| --- | --- | --- |
| EUB-8-f | AGAGTTTGATCMTGGCTCAG | Bakke et al. (2011) |
| 907-r | CCGTCAATTCMTTTGAGTTT | Muyzer et al. (1995) |
| Primers (2^nd^ round, V3-V4 region) |  |  |
| PCR1F_460 | CTTTCCCTACACGACGCTCTTCCGATCTACGGRAGGCAGCAG | Carmona-Martinez et al. (2015) |
| PCR1R_460 | GGAGTTCAGACGTGTGCTCTTCCGATCTTACCAGGGTATCTA | Carmona-Martinez et al. (2015) |

**References**

Bakke I, De Schryver P, Boon N, Vadstein O: **PCR-based community structure studies of Bacteria associated with eukaryotic organisms: A simple PCR strategy to avoid co-amplification of eukaryotic DNA**. *J Microbiol Meth* 2011, **84**:349-351.

Carmona-Martinez AA, Trably E, Milferstedt K, Lacroix R, Etcheverry L, Bernet N: **Long-term continuous production of H_2_ in a microbial electrolysis cell (MEC) treating saline wastewater**. *Water Res* 2015, **81**:149-156.

Muyzer G, Teske A, Wirsen C, Jannasch H: **Phylogenetic relationships of *Thiomicrospira* species and their identification in deep-sea hydrothermal vent samples by denaturing gradient gel electrophoresis of 16S rDNA fragments**. *Arch Microbiol* 1995, **164**:165-172.
